# Quantitative Analysis of Single and Mix Food Antiseptics Basing on SERS Spectra with PLSR Method

**DOI:** 10.1186/s11671-016-1507-5

**Published:** 2016-06-14

**Authors:** Mengjing Hou, Yu Huang, Lingwei Ma, Zhengjun Zhang

**Affiliations:** State Key Laboratory of New Ceramics and Fine Processing, School of Materials Science and Engineering, Tsinghua University, Beijing, 100084 People’s Republic of China; Key Laboratory of Advanced Materials (MOE), School of Materials Science and Engineering, Tsinghua University, Beijing, 100084 People’s Republic of China

**Keywords:** Food antiseptics, Surface-enhanced Raman scattering, Partial least squares regression, Quantitative analysis, Mixture analysis

## Abstract

Usage and dosage of food antiseptics are very concerned due to their decisive influence in food safety. Surface-enhanced Raman scattering (SERS) effect was employed in this research to realize trace potassium sorbate (PS) and sodium benzoate (SB) detection. HfO_2_ ultrathin film-coated Ag NR array was fabricated as SERS substrate. Protected by HfO_2_ film, the SERS substrate possesses good acid resistance, which enables it to be applicable in acidic environment where PS and SB work. Regression relationship between SERS spectra of 0.3~10 mg/L PS solution and their concentration was calibrated by partial least squares regression (PLSR) method, and the concentration prediction performance was quite satisfactory. Furthermore, mixture solution of PS and SB was also quantitatively analyzed by PLSR method. Spectrum data of characteristic peak sections corresponding to PS and SB was used to establish the regression models of these two solutes, respectively, and their concentrations were determined accurately despite their characteristic peak sections overlapping. It is possible that the unique modeling process of PLSR method prevented the overlapped Raman signal from reducing the model accuracy.

## Background

Quantitative analysis of trace chemicals has been highly focused in the fields such as food science [1, 2], environmental science [3, 4], and biology [5, 6]. With the assistance of surface-enhanced Raman scattering (SERS) effect, Raman spectrum of trace analyte could be detected. Moreover, the intensity of Raman peak could reflect the amount of analyte, which makes quantitative analysis feasible [7]. The most commonly used method appears that calculating the relationship between the intensity of a strong characteristic peak and solution concentration [8, 9]. However, this method is unstable, as it is more possible for the intensity of one peak being influenced by some objective factors. Therefore, a more accurate method called “partial least squares regression” (PLSR) has been preferred. Intensity data of all characteristic peaks would be employed in modeling process, and redundant information contained in spectra would be eliminated through principal component analysis of observing matrix. In recent years, quantitative analysis of trace analyte by PLSR analysis of SERS spectra has been adopted by many researchers [10–12].

Food antiseptics are chemicals which could inhibit bacteria and extend the food’s shelf life, and their content is usually small. Only if with proper content, antiseptics could perform best; besides, excess of these chemicals may harm people’s health [13–16]. Hence, quantitative analysis of antiseptics basing on their SERS spectra is of great importance. Potassium sorbate (PS) and sodium benzoate (SB) are both the most commonly used food antiseptics, and they could only work in acidic condition [17–19]. Their content in food should be controlled accurately. Furthermore, as they inhibit different kinds of bacteria, sometimes both of them should be added into food meanwhile [20, 21]. It is obvious that concentrations of the single solute solution as well as the mixture solution both need to be determined in practice.

In this research, trace food antiseptics were detected employing SERS effect, and their concentrations were determined by analyzing SERS spectra with PLSR method. The regression relationship between the concentration and SERS spectra of a series of PS solution performs well in predicting concentration of PS solution samples. As to the mixture solution of PS and SB, even if their characteristic peaks overlapped, the two PLSR models of them both appeared accurate and stable. To detect the Raman spectra of trace antiseptic solution, HfO_2_ ultrathin film-coated Ag nanorod array was fabricated as SERS substrate, and this kind of substrate possessed the property of acid resistance, which enables it to be applicable in acid environment where PS and SB could work in practice.

## Methods

### Fabrication of HfO_2_ Ultrathin Film-Coated Ag Nanorod Array SERS Substrate

Ag nanorod (NR) array was employed as SERS substrate, and HfO_2_ ultrathin film was deposited on the surface of NRs to protect Ag from becoming oxidized. Ag NRs were fabricated with a DZS-500 electronic beam evaporation system (SKY Technology Development Co., Ltd. Chinese Academy of Sciences). The background vacuum was 5 × 10^−6^ Pa, aiming at avoiding oxidation of Ag. To obtain discrete nanostructures on wafer, glancing angle deposition (GLAD) method was adopted. The wafer was placed above the crucible obliquely and the incident angle of Ag beam was 86°.

Then, the Ag NRs were coated with HfO_2_ ultrathin film through atomic layer deposition (ALD) system (MNT-100, Wuxi MNT Micro and Nanotech Co.). Precursors to synthesize HfO_2_ were tetrakis (dimethylamino) hafnium and water. In an 80 °C vacuum chamber, the Ag NR sample was made thermal equilibrium. Tetrakis (dimethylamino) hafnium was pumped in at first, and after the residues were cleared, water was pumped in, followed by residues cleared again. Thus, film of particular thickness was formed.

### Characterization of SERS Substrate

Morphology of the HfO_2_ ultrathin film-coated Ag NR SERS substrate was characterized with scanning electron microscope (Merlin VP Compact) and high-resolution transmission electron microscope (JEOL-2100F).

### Preparation and Adsorption of Analyte Solution

4-mercaptobenzoic acid (4-Mba) (J&K Scientific Ltd) was employed as probe molecule to measure the SERS effect of the HfO_2_-coated Ag NR substrate. 1 × 10^−5^, 1 × 10^−6^, 1 × 10^−7^, 1 × 10^−8^, and 1 × 10^−9^ M ethanol solution of 4-Mba was prepared, and the SERS substrates were immersed in the solution for 1 h to make 4-Mba molecules adsorbed. Then, the substrates were rinsed with ethanol.

PS powder was dissolved in deionized water and diluted with ethanol to the concentration of 0.3, 0.5, 0.8, 1, 3, 5, 8, and 10 mg/L, respectively. Mixture solution of PS and SB with the composition as Table 1 shows was also prepared. A 5-μL solution was dropped on SERS substrate each time to make these analyte molecules adsorbed.

**Table 1:** Compositions of PS and SB mixture solution

Number	PS concentration (mg/L)	SB concentration (mg/L)	Number	PS concentration (mg/L)	SB concentration (mg/L)
1	0	0	19	0	10
2	1	0	20	1	10
3	5	0	21	5	10
4	10	0	22	10	10
5	50	0	23	50	10
6	100	0	24	100	10
7	0	1	25	0	50
8	1	1	26	1	50
9	5	1	27	5	50
10	10	1	28	10	50
11	50	1	29	50	50
12	100	1	30	100	50
13	0	5	31	0	100
14	1	5	32	1	100
15	5	5	33	5	100
16	10	5	34	10	100
17	50	5	35	50	100
18	100	5	36	100	100

### Measurements of SERS Spectra

Raman spectra of the analytes were measured by a micro-Raman spectrometer (i-Raman Plus, B&W TEK Inc.). A 785-nm laser was used as excitation source, and its power was 300 mW. The diameter of beam spot was 85 μm.

### Measurements of Absorption Spectrum

Absorption spectrum of the SERS substrate was calculated by subtracting the reflectance from 100 %, and the reflectance spectrum was measured with the R1 angle-resolved spectroscopy system (Idea Optics Co.).

## Results and Discussion

**Fig. 1:**
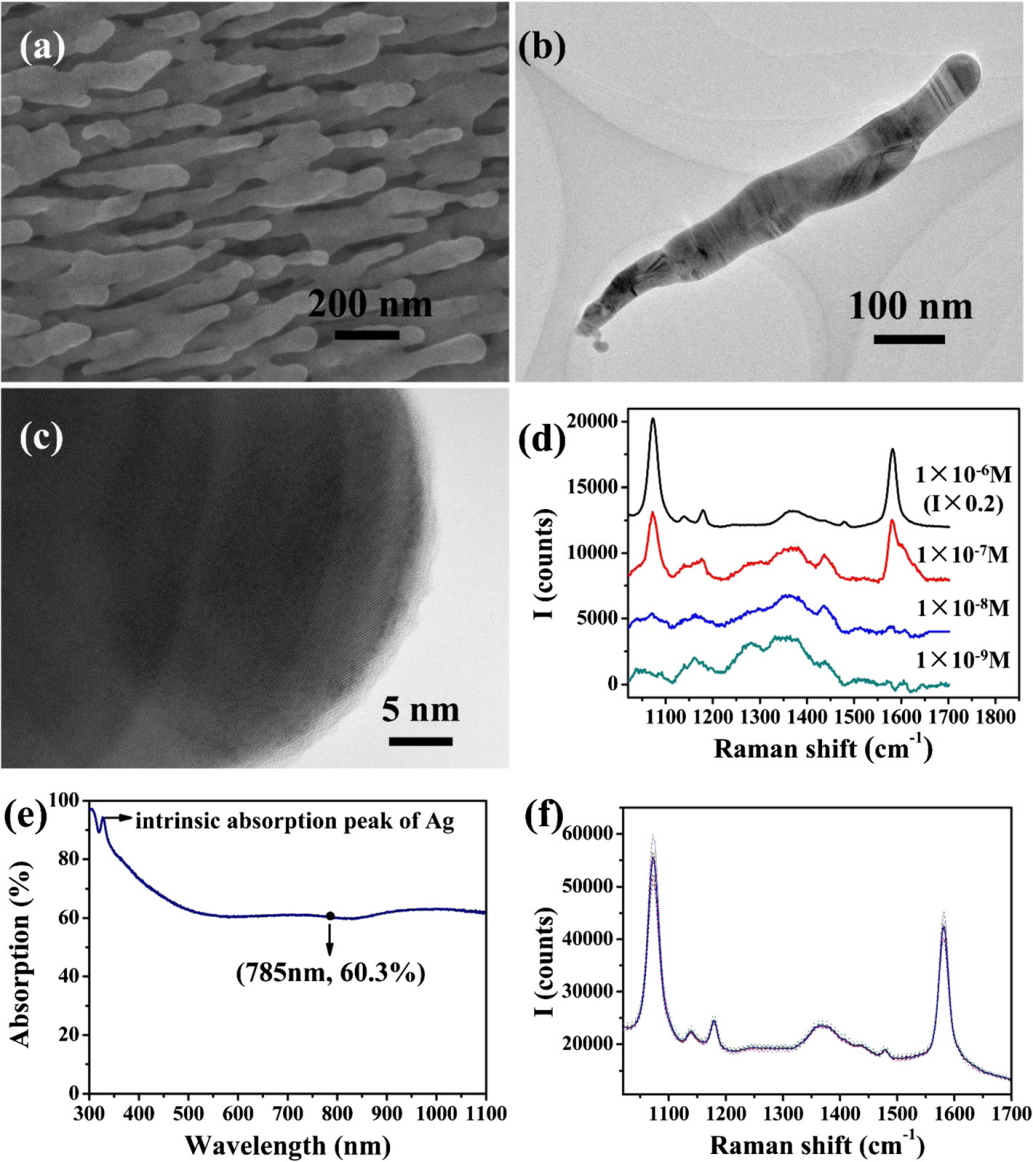
**a** SEM morphology of the HfO_2_ ultrathin film-coated Ag NR SERS substrate; **b** TEM morphology of a HfO_2_ ultrathin film-coated Ag NR; **c** high-resolution TEM of HfO_2_ ultrathin film outside the Ag NR; **d** Raman spectra of 1 × 10^−6^, 1 × 10^−7^, 1 × 10^−8^, and 1 × 10^−9^ M 4-Mba adsorbed on SERS substrate; **e** absorption spectrum of the HfO_2_ ultrathin film-coated Ag NR SERS substrate; **f** the *dotted lines* are Raman spectra of 1 × 10^−6^ M 4-Mba measured on 10 different sites randomly selected on SERS substrate, and the *solid line* is the average spectrum of them

**Fig. 2:**
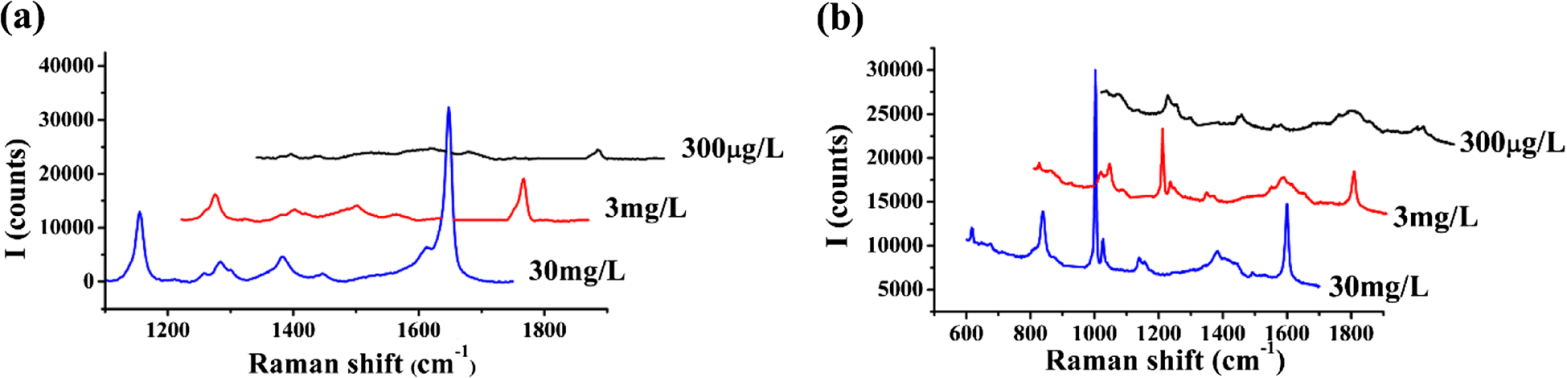
**a** SERS spectra of 30, 3, and 300 μg/L PS; **b** SERS spectra of 30, 3, and 300 μg/L SB

HfO_2_ ultrathin film-coated Ag NR array was used as SERS substrate in this research. As Fig. 1a shows, oblique NRs separate with each other, and the diameters of these NRs range from 30 to 80 nm. Observed in Fig. 1b, c, the NRs are about 700 nm in length, and thickness of HfO_2_ film is about 9 Å. The film coats the Ag NRs completely with a uniform thickness, which could isolate oxygen effectively. However, film out of Ag would weaken the SERS effect, for the analyte molecule could not reach the surface of Ag [22, 23]. According to the electromagnetic mechanism, intensity of surface electric field would decrease sharply with the increase of the distance from the metal surface [24–26]. Separation of 4-Mba molecules and Ag also prevented Ag—S bond from forming, which would also lead to weaker Raman signal [27, 28]. As to this SERS substrate, according to Fig. 1d, the limit of detection for 4-Mba was 1 × 10^−8^ M, which was quite satisfactory. SERS effect remained strong, for the HfO_2_ film was deposited very thin, and its thickness could be controlled precisely, benefitting from the self-limiting nature of the ALD method [29–31]. The SERS substrate is also applicable in a quite wide wavelength range. As the absorption spectrum (Fig. 1e) shows, in the wavelength scale from 450 to 1100 nm, absorption of the SERS substrate keeps around 60 %. At 785 nm, the absorption is 60.3 %, which indicates that the 785-nm laser employed by the Raman spectrometer is suitable for the SERS substrate. Besides, the reproducibility of the SERS substrate is quite good, which could be verified in Fig. 1f. The SERS spectra of 1 × 10^−6^ M 4-Mba measured on 10 different sites randomly selected on the substrate are similar, and none of them shows obvious differences from the average spectrum. The SERS substrate performed sensitively in detecting PS and SB. The limit of detection (LOD) of these two kinds of antiseptics is both 300 μg/L, as Fig. 2a, b shows, and this concentration data is much lower than the general dosage of the antiseptics in food. Therefore, the SERS substrate could meet the demand of detecting PS and SB in practice.

**Fig. 3:**
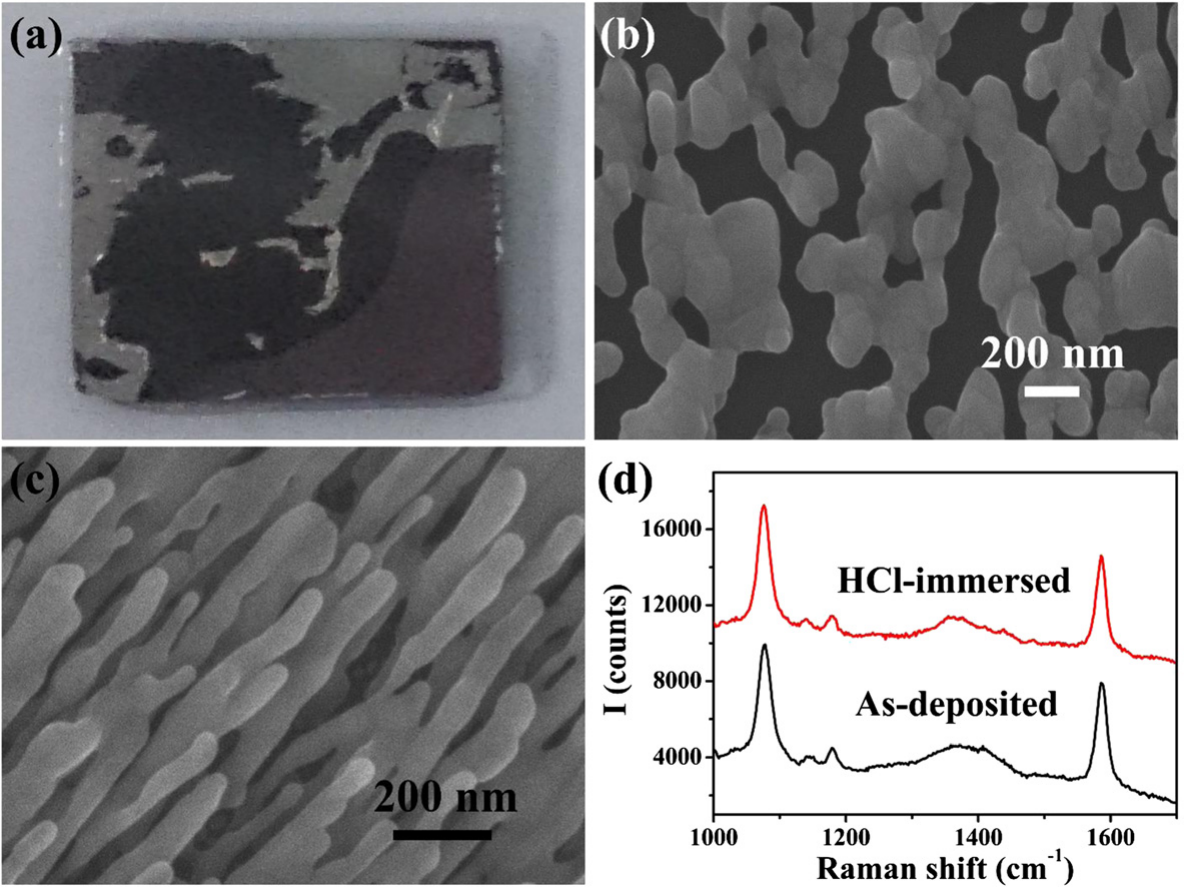
**a** Ag NR array on wafer after immersed in 10 mM HCl; **b** SEM morphology of Ag NR array after immersion in 10 mM HCl; **c** SEM morphology of the HfO_2_ ultrathin film-coated Ag NRs after immersion in 10 mM HCl; **d** Raman spectra of 1 × 10^−5^ M 4-Mba adsorbed on as-deposited and 10 mM HCl immersed HfO_2_ ultrathin film-coated Ag NR SERS substrates

In consideration that PS and SB could only work in acidic condition, it is important in practical detecting process that the SERS substrate has property of acid resistance. HfO_2_ does not react with acids such as HCl, HNO_3_, and citric acid, hence it would protect Ag SERS substrate from failure in acid environment. Figure 3a shows that after immersion in 10 mM HCl, Ag NRs detached from the wafer. The morphology of residual Ag NRs observed with SEM (Fig. 3b) shows that the NRs had been dissolved and “hot spots” almost disappeared. In contrast, morphology of HfO_2_-coated Ag NRs remained unchanged after immersion in 10 mM HCl, as Fig. 3c shows, which ensured that the substrate’s SERS effect never decreased consequently. According to Fig. 3d, peak intensities of 4-Mba measured with as-deposited HfO_2_-coated Ag NR substrate and substrate which was HCl-immersed and water-rinsed are nearly the same, which proves that the SERS substrate possesses great acid resistance.

**Fig. 4:**
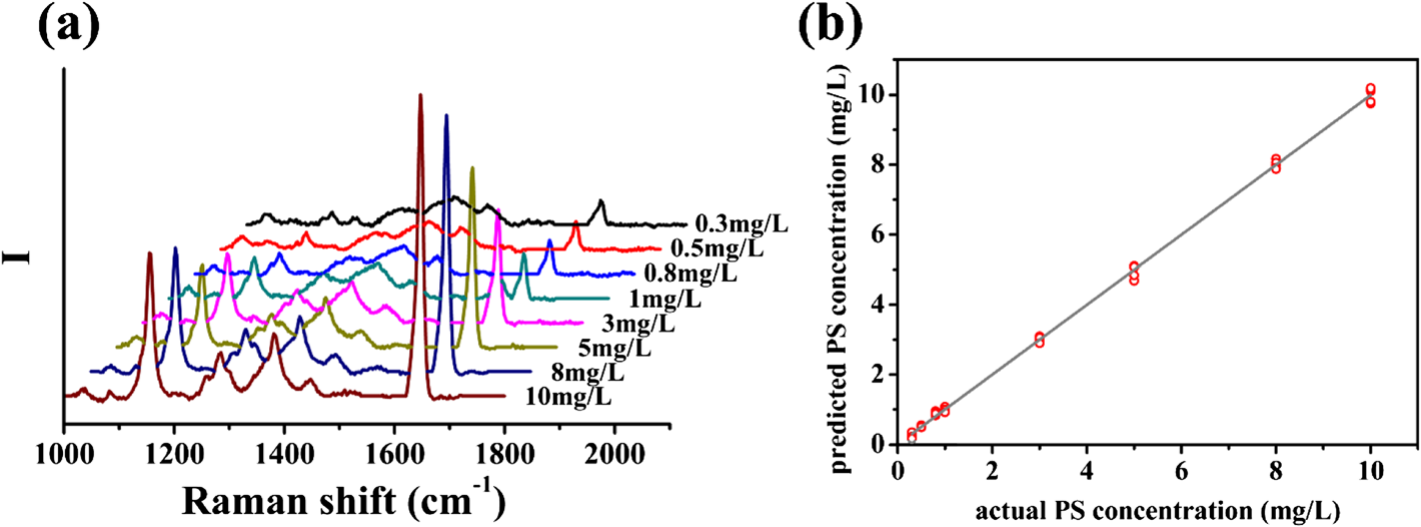
**a** average Raman spectra of 0.3~10 mg/L PS adsorbed on the SERS substrates; **b** PS concentration predicted by the PLSR model established with Raman spectra of 0.3~10 mg/L PS. Five spectra of each sample were measured on positions selected randomly, and the integration time was 20 s

With the HfO_2_ ultrathin film-coated Ag NR SERS substrate, Raman spectra of 0.3~10 mg/L PS solution was measured, as Fig. 4a shows. Intensity of characteristic peaks increased along with the solution concentration. PLSR method was employed here to establish the regression relation between SERS spectra and solution concentration. Principal components of observing matrix composed of centered SERS spectra were extracted in sequence to maximize its covariance with solution concentration, and the regression coefficient between observing matrix and concentration was determined. To calculate the solution concentration corresponding to a SERS spectrum, the centered spectrum data would be multiplied with the regression coefficient, followed by adding the average concentration data of all calibration samples. The PLSR models were established with BWIQ® chemometrics software (B&W TEK Inc.) The concentrations predicted by the PLSR model are quite close to the actual concentrations as plotted in Fig. 4b. *R*^2^ of the linear relation between two series of concentrations is 0.999, and root mean square error (RMSE) of these calibration data is 1.114 mg/L, which reflects the perfect accuracy of the model. To verify the feasibility of the model, SERS spectra of another series of 0.3~10 mg/L PS solution were analyzed as test samples. With the loading matrix calculated in the PLSR model, concentration of PS solution for test was obtained. *R*^2^ of the linear relation between predicted and actual concentration is 0.998, and RMSE of these test data is 1.898 mg/L, which indicates that the model is stable and applicable.

In many kinds of food, PS and SB are usually compounded, for their functions are different. Besides, their solubility in commonly used solvents is similar, which makes quantitative analysis of their mixture solution essential. Herein, PLSR analyses specific to PS and SB were carried out respectively.

**Fig. 5:**
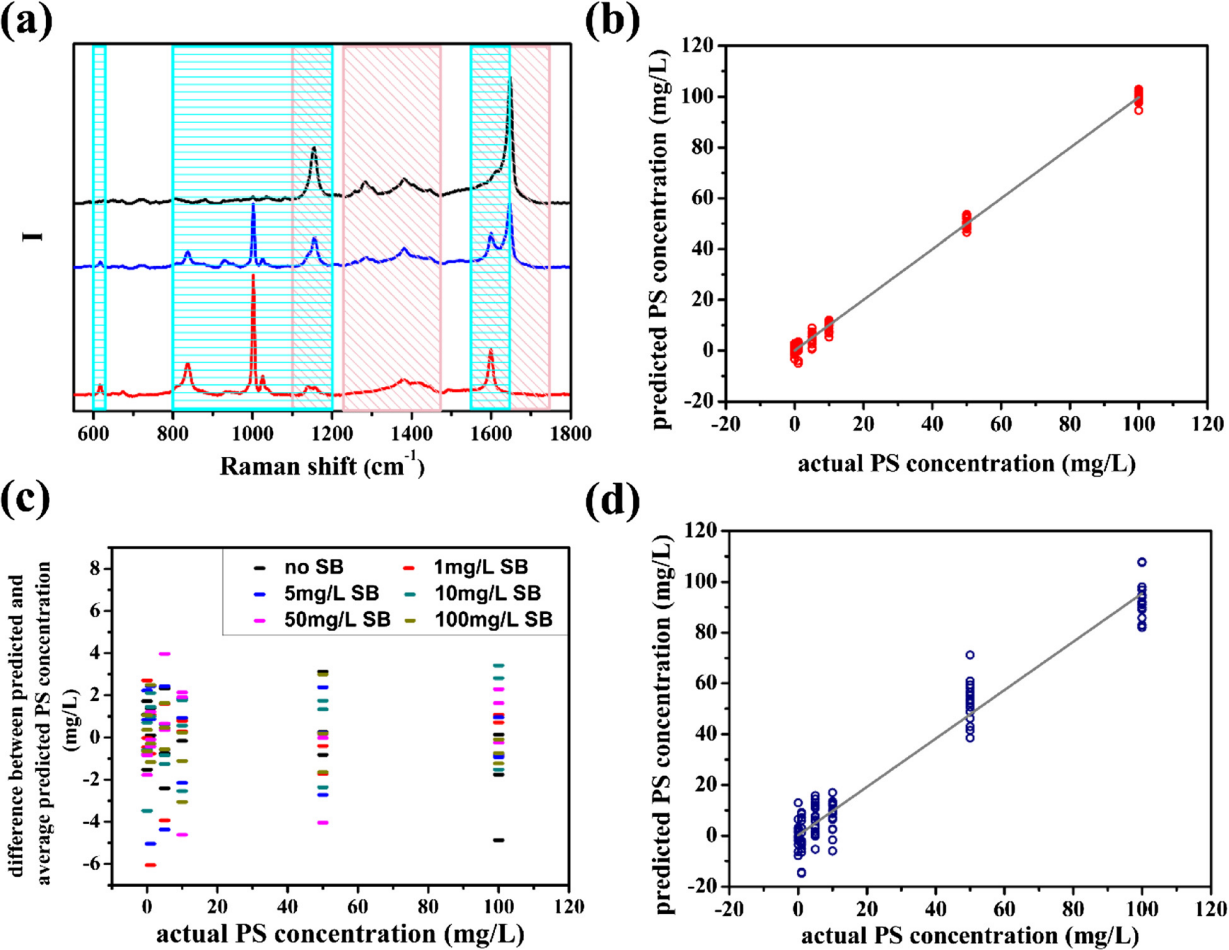
**a** The *black*, *blue*, and *red lines* represent Raman spectra of PS solution, mixture solution contained 10 mg/L PS and 10 mg/L SB, and SB solution measured with SERS substrates, respectively, and the *pink* and *blue rectangles* mark the characteristic peak sections of PS and SB employed to calibrate PLSR model, respectively; **b** PS concentration predicted by the PLSR model established with Raman spectrum data corresponding to PS characteristic peaks; **c** difference between predicted PS concentration of each mixture solution and average predicted PS concentration of solution with the same PS concentration; **d** PS concentration in test solution with the composition as Table 1 shows predicted by the PLSR model corresponding to PS

To measure PS concentration in the 36 kinds of solution listed in Table 1, intensity of characteristic peak sections of PS in these spectra all acted as calibration data. It could be found in SERS spectra of PS and SB solution, and mixture solution of them, such as the ones shown in Fig. 5a that characteristic peak sections of PS and SB overlapped. While establishing the quantitative model of PS, the SB signal acted as interference signal. According to Table 1, SB concentration varied from 0 to 100 mg/L among the samples which had the same PS concentration, hence the SB Raman signal which overlapped with PS signal also varied in a very large scale. As a result, the SB signal could not be subtracted as background. However, as Fig. 5b shows, predicted PS concentrations agree well with the actual concentrations. *R*^2^ of the linear relation between predicted and actual PS concentration is 0.997, and RMSE of these test data is 1.950 mg/L. Moreover, the predicted PS concentrations in mixtures with different SB concentrations are almost equal; as shown in Fig. 5c, their differences to average predicted PS concentration of all solution with the same PS concentration are similar. That is to say, Raman signal corresponding to SB in spectra did not interfere the quantitative analysis for PS. This positive phenomenon may be originated from the unique modeling process of PLSR method [32, 33]. Loading vector corresponding to each principal component should be determined based on the criteria that maximizing variance of latent variable as well as correlation coefficient between latent variable and dependent variable at the same time, which could be realized by maximizing the covariance of the latent variable and dependent variable. Thus, principal components extracted from observing matrix not only represent the most difference of the calibration spectra, but also relate to the concentration data as closely as possible. Therefore, even if Raman signal of SB was contained in the calibration spectra of PS quantitative model and varies in a large range, the information which related to the PS concentration most closely would be extracted, and information about SB would be removed, thus the established model could predict PS concentration in mixture accurately. The PS quantitative model was then tested by another series solution also with the composition as Table 1 shows. The predicted PS concentration plotted in Fig. 5d obtained a RMSE of 7.034 mg/L, and the *R*^2^ of the linear relation between predicted and actual concentration is 0.982.

**Fig. 6:**
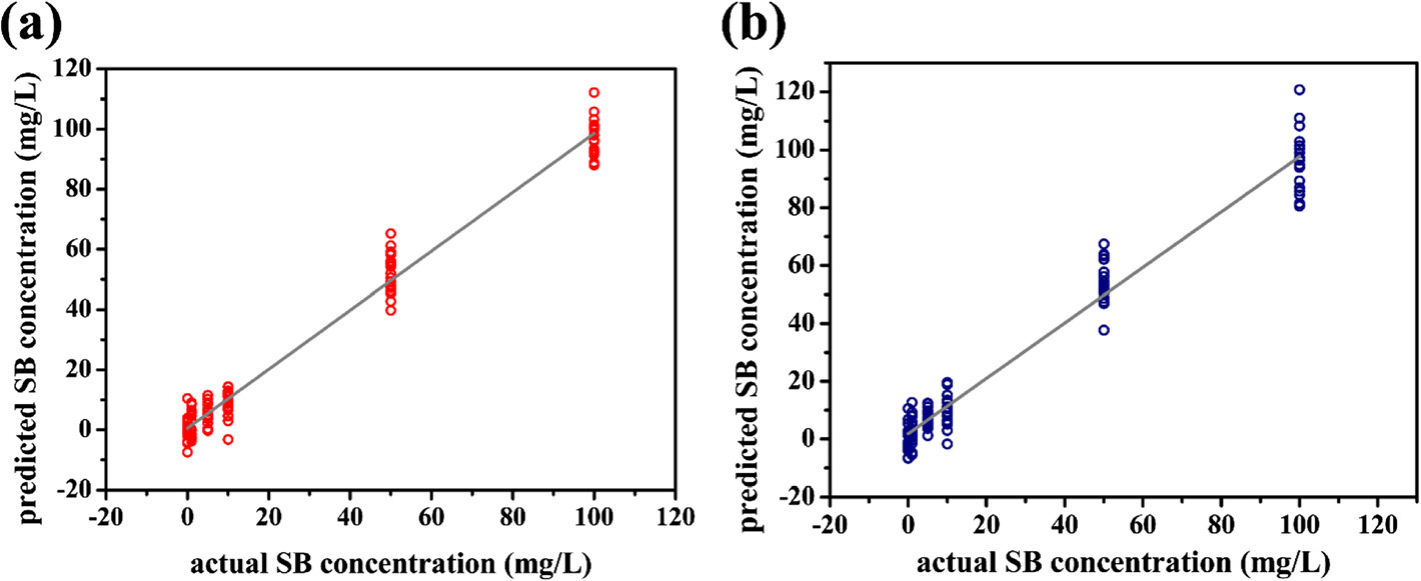
**a** SB concentration predicted by the PLSR model established with Raman spectra data corresponding to SB characteristic peaks; **b** SB concentration in test solution with the composition as Table 1 shows predicted by the PLSR model corresponding to SB

Basing on the same principle, quantitative analysis model of SB were also established with spectrum intensity data within the characteristic peak sections of SB, and there was also PS Raman signal in the sections. The predicted SB concentrations meet the actual concentrations well as shown in Fig. 6a. *R*^2^ of the linear relation between two series of concentrations is 0.981, and RMSE of these calibration data is 5.060 mg/L. The predicting result was also never interfered by PS signal in mixtures’ spectra. Employing the quantitative model, the series of SERS spectra of mixture solution for test whose compositions were the same as the calibration samples were analyzed, and SB concentrations were determined, as Fig. 6b shows. *R*^2^ of the linear relation between predicted and actual concentration is 0.964, and RMSE of these data is 6.966 mg/L. The calibration and validation results indicated that PLSR method performed well in analyzing SERS spectrum of mixture solution, and this quantitative method should be applicable in food antiseptic mixture analysis.

## Conclusions

Potassium sorbate and sodium benzoate which are most commonly used antiseptics in acidic food were detected with a kind of novel SERS substrate. The substrate was acid resistant because of HfO_2_ ultrathin film which coating the Ag NRs completely would act as protective shell while weakened the SERS effect as slightly as possible. Therefore, the SERS substrate may bear acidic environment when detecting trace PS and SB, and this performance met the demands of practical detection properly.

Quantitative analysis of trace PS solution and mixture solution of PS and SB was carried on with PLSR method. SERS spectra of 0.3~10 mg/L PS solution were employed as calibration data to establish the regression model, and the PS concentration was predicted accurately with the model. Spectrum data of characteristic peak sections corresponding to PS and SB was selected respectively to establish PLSR model of the two kinds of solutes in mixture solution. Even if their peak sections overlapped, the two regression models are both accurate and stable, without interfered by the Raman signal coming from the other solute. The reason may lie in the unique modeling process of PLSR method. The satisfactory quantitative results demonstrated the feasibility of the method in single and mix food antiseptics analysis.
